# 

**Published:** 1875-02

**Authors:** 


CONTENTS FOR FEBRUARY, 1875.

| | | | |
|---|---|---|---|
| Fire on the Hearth | 29 | Hospital Reform | 41 |
| Medical Profession | 31 | Ship Biscuits | 42 |
| Covering for the Feet | 35 | The Helping Hand | 43 |
| Floral Guide | 36 | The Best Society | 44 |
| Human Weights | 37 | Unappreciated | 45 |
| Girls behind Counters | 37 | Save your Children | 46 |
| Saving Fuel | 38 | Plants in Sleeping Rooms | 47 |
| Human Stove | 39 | Public Speaking | 48 |
| Aches and Pains | 39 | Hall's Medical Adviser | 49 |
| Wall Paper | 39 | Sphynx | 52 |
| Sciatica | 40 | Joy to the World | 53 |
| Flowers a Remedy | 41 | Family Health | 55 |

